# Predicted distribution of the glass sponge *Vazella pourtalesi* on the Scotian Shelf and its persistence in the face of climatic variability

**DOI:** 10.1371/journal.pone.0205505

**Published:** 2018-10-24

**Authors:** Lindsay Beazley, Zeliang Wang, Ellen Kenchington, Igor Yashayaev, Hans Tore Rapp, Joana R. Xavier, Francisco Javier Murillo, Derek Fenton, Susanna Fuller

**Affiliations:** 1 Department of Fisheries and Oceans, Bedford Institute of Oceanography, Dartmouth, Nova Scotia, Canada; 2 Department of Biological Sciences and K.G. Jebsen Centre for Deep-Sea Research, University of Bergen, Bergen, Norway; 3 CIIMAR—Interdisciplinary Centre of Marine and Environmental Research, University of Porto, Avenida General Norton de Matos, Matosinhos, Portugal; 4 Oceans North, Halifax, Nova Scotia, Canada; University of Rhode Island, UNITED STATES

## Abstract

Emerald Basin on the Scotian Shelf off Nova Scotia, Canada, is home to a globally unique aggregation of the glass sponge *Vazella pourtalesi*, first documented in the region in 1889. In 2009, Fisheries and Oceans Canada (DFO) implemented two Sponge Conservation Areas to protect these sponge grounds from bottom fishing activities. Together, the two conservation areas encompass 259 km^2^. In order to ascertain the degree to which the sponge grounds remain unprotected, we modelled the presence probability and predicted range distribution of *V*. *pourtalesi* on the Scotian Shelf using random forest modelling on presence-absence records. With a high degree of accuracy the random forest model predicted the highest probability of occurrence of *V*. *pourtalesi* in the inner basins on the central Scotian Shelf, with lower probabilities at the shelf break and in the Fundian and Northeast Channels. Bottom temperature was the most important determinant of its distribution in the model. Although the two DFO Sponge Conservation Areas protect some of the more significant concentrations of *V*. *pourtalesi*, much of its predicted distribution remains unprotected (over 99%). Examination of the hydrographic conditions in Emerald Basin revealed that the *V*. *pourtalesi* sponge grounds are associated with a warmer and more saline water mass compared to the surrounding shelf. Reconstruction of historical bottom temperature and salinity in Emerald Basin revealed strong multi-decadal variability, with average bottom temperatures varying by 8°C. We show that this species has persisted in the face of this climatic variability, possibly indicating how it will respond to future climate change.

## Introduction

Deep-sea sponge-dominated communities have gained increasing attention in recent years from both an ecological and conservation perspective. Growing evidence not only suggests that these habitats are widely distributed across the deep sea globally [1–2], but that they also play key functional roles, directly or indirectly, in delivering a number of ecosystem goods and services. This includes, but is not limited to, habitat-provision [3–10], biodiversity enhancement [11–15], and biogeochemical cycling [16–20] (see also the review by [2]).

Glass sponges (Class Hexactinellida) are among the most ancient of the extant metazoans, with both paleontological and molecular data suggesting their establishment in the late Proterozoic or earlier [21–22]. According to the World Porifera Database there are over 600 hexactinellid species globally, of which roughly 70 occur in the North Atlantic [23]. Glass sponges are distributed along a large depth gradient, but are most abundant and diverse in the bathyal zone, i.e., at depths of 200–3000 m [24]. They are usually sparsely distributed on various types of substrate. However, in some areas a few species form dense populations, either monospecifically or as part of a more diverse, multispecies community. Examples in the North Atlantic include the monospecific grounds of *Pheronema carpenteri* in the northeast Atlantic between 650–2000 m depth (e.g., [4,25]); *Poliopogon amadou* on the Great Meteor seamount at 2700 m depth [26]; *Nodastrella asconemaoida* on the bathyal coral reefs of Rockall Bank at 580 m depth [27]; and the species *Asconema foliata* and *Schaudinnia rosea* found in the multispecific tetractinellid sponge grounds on the Flemish Cap and Grand Banks [28], in the Denmark Strait [29], and along the Arctic Mid-Ocean Ridge [30–31].

**Fig 1:**
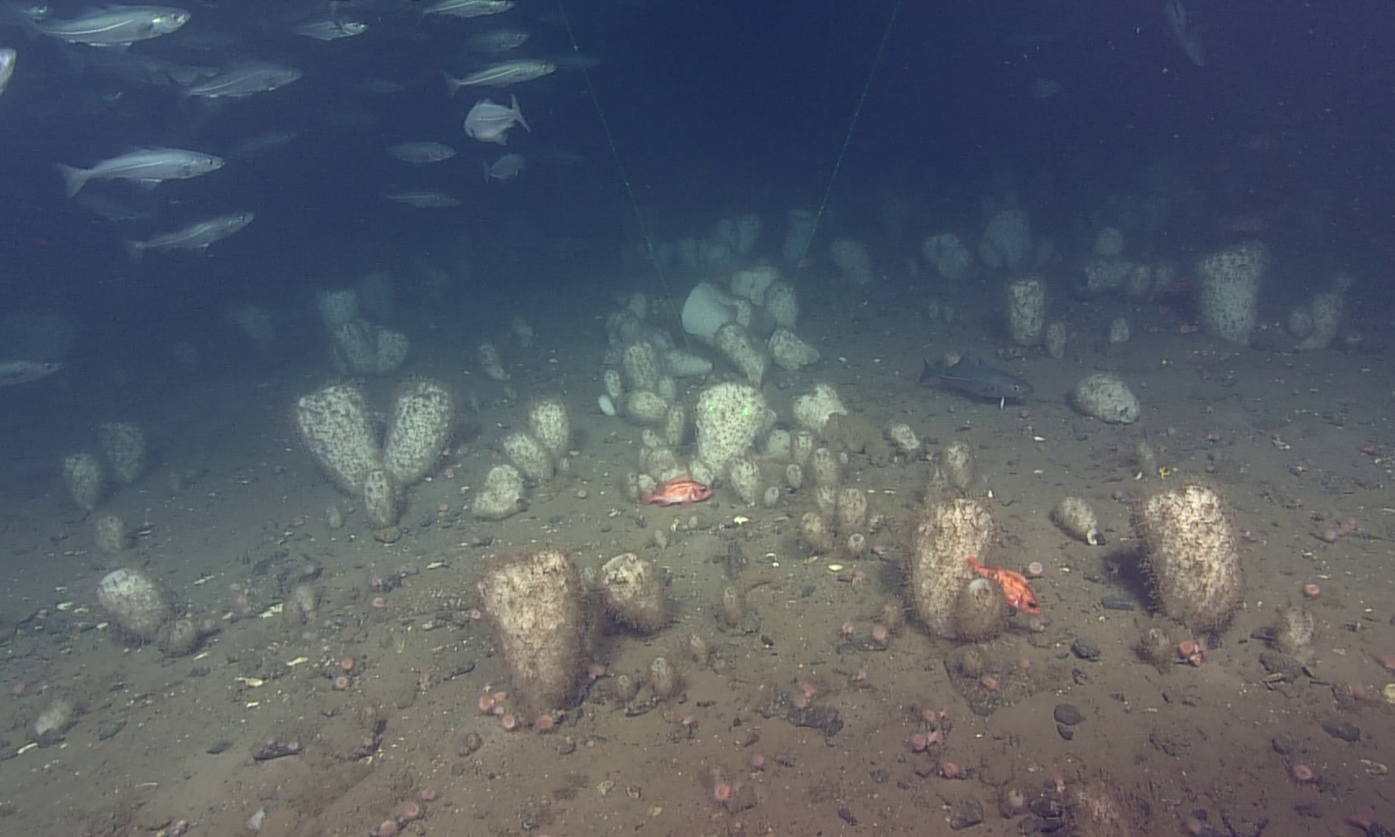
Glass sponge *Vazella pourtalesi* [32] in Emerald Basin, Nova Scotia, Canada. Image is a framegrab from video collected using the ROV ROPOS in 2017. Depth is 195 m, location is 43°52.1225 N, -63°2.8945 W.

A lesser known dense population of glass sponges is formed by the rosellid *Vazella pourtalesi* [32] on the Scotian Shelf off Nova Scotia, Canada (Fig 1). *V*. *pourtalesi* was originally described by Schmidt [32] as *Holtenia pourtalesii* from material collected in the Florida Keys between 282 and 583 m depth. The same author described a second species (*H*. *saccus*) for the same area, but re-examination of the type material of both species provided evidence of their conspecific status [33]. A few years after the first description of this species was made, Honeyman [34] described the collection of a hexactinellid sponge in the late 1880’s from the Scotian Shelf off Nova Scotia, Canada that resembled a “Cap of Liberty”, “brought up from a depth of 80 fathoms, at a distance of 40 miles south of Sambro”. The collection was regarded as unique at the time. Over a century later in 2001, the specimen was identified as *V*. *pourtalesi* by glass sponge taxonomist H.M. Reiswig [35], who reinstated the genus in his 1996 revision [33]. The occurrence of a dense aggregation of these sponges on the Scotian Shelf (referred to as the ‘*Vazella* sponge grounds’ herein) was noted in the late 1990’s during interviews with local fishermen that were conducted to ascertain the types of fish and invertebrates found on the seabed off Nova Scotia [36]. Fisherman described these sponges as “Russian Hats” that filled the nets when fishing for pollock and redfish [36]. In 2001, the first *in situ* video footage of the *Vazella* sponge grounds in Emerald Basin was collected. The densities observed on the seabed (up to 16 individuals per m^2^), its relatively shallow depth distribution (75–275 m), and its large size (up to 110 cm in height) relative to previously examined specimens (up to 9.2 cm in height; [35]) rendered this aggregation globally unique. Aside from the specimens collected off Florida, to date *V*. *pourtalesi* has only been reported from one other location, the Azores, where a single specimen was collected amongst several specimens of *Pheronema carpenteri* at 845 m depth [37].

### Conservation status

The *Vazella* sponge grounds on the Scotian Shelf are located in traditional groundfish fishing grounds [36], with the majority of activity occurring from the pollock, silver hake, redfish, and halibut bottom trawl fisheries. As a result, high bycatch of *V*. *pourtalesi*, with records upwards of 9000 kg, has been reported [36,38]. Fisheries and Oceans Canada (DFO) took the first step towards elevating the conservation status of the population of *V*. *pourtalesi* in 2004, when an area commonly referred to as ‘The Patch’ by local fisherman was recognized as an ecologically significant area. This recognition was based on the rarity/uniqueness of the *Vazella* sponge grounds present in the area and their fragility/sensitivity to fishing impacts [39]. Information from fishermen, bycatch records from commercial fisheries, and *in situ* scientific observations were used to delineate hotspots of *V*. *pourtalesi* in The Patch and nearby areas in Emerald Basin and Sambro Bank [38]. This ecologically significant area was later modified in its extent to encompass a larger portion of Emerald Basin and was formally identified as an Ecologically and Biologically Significant Area (EBSA) by DFO in 2006 [40]. Under the directive of the United Nations General Assembly (UNGA) Resolution 61/105, this species also received status as a vulnerable marine ecosystem (VME) indicator in 2008 by the Northwest Atlantic Fisheries Organization (NAFO; [41]).

In 2009, DFO introduced the Policy for Managing the Impacts of Fishing on Sensitive Benthic Areas (SBA; http://www.dfo-mpo.gc.ca/reports-rapports/regs/sff-cpd/benthi-eng.htm) as part of the Sustainable Fisheries Framework policy suite, aimed at achieving a more ecosystem-based approach to fisheries management. The SBA Policy was created for the purpose of aiding DFO in “managing fisheries to mitigate impacts of fishing on sensitive benthic areas or avoid impacts of fishing that are likely to cause serious or irreversible harm to sensitive marine habitat, communities and species”, and is the main tool used by DFO to mirror in domestic waters its commitment under the UNGA 61/105 to identify and protect vulnerable marine ecosystems on the high seas from bottom-contact fishing. To assist with the identification and mapping of SBAs, DFO held a national advisory process (NAP) that included both scientists and stakeholders on the ‘Occurrence, Susceptibility to Fishing, and Ecological Function of Corals, Sponges, and Hydrothermal Vents in Canadian Waters’ [42]. The uniqueness of the *Vazella* sponge grounds on the Scotian Shelf, the biogenic habitat it forms, and its fragility and risk of serious or irreversible harm from fishing qualified it for protection under the SBA Policy, and as a result, the habitat occupied by *V*. *pourtalesi* emerged during the 2010 NAP as a clear conservation priority for the Department. Through kernel density estimation (KDE) analysis of research vessel trawl catch this habitat was determined to occupy an area of ~8,000 km^2^ [43]. This approach does not take into account environmental variables and is based solely on the co-location of catches with high sponge weights, and the transition between the area occupied by those catches and those surrounding catches outside of the sponge grounds (see [44] for description). Following the NAP, the Groundfish Enterprise Allocation Council (GEAC) implemented a voluntary closure to protect the sponges from mobile gear (http://www.dfo-mpo.gc.ca/oceans/publications/cs-ce/page09-eng.html). In 2013, two bottom fishery closure areas were designated by DFO to protect the significant concentrations of *V*. *pourtalesi* in Emerald Basin, representing the first application of the SBA Policy in Canadian waters. The design of the conservation areas was informed by a working group that included fishing representatives of active fleets in the area. The Emerald Basin *Vazella* Conservation Area is 197 km^2^ in size, and represents a modified version of the original boundaries of the voluntary closure implemented by GEAC in 2010. A second area 62 km^2^ in size, termed the Sambro Bank *Vazella* Conservation Area, was implemented over a second significant concentration of *Vazella* in order to protect the area from bottom contact fishing gears and to provide replication to aid population recovery. These two conservation areas protect only ~3% of the total 8,000 km^2^ habitat occupied by the *Vazella* sponge grounds (as determined from KDE analysis).

### Distribution modelling and climatological setting

As supporting research for a recent re-evaluation of the location of significant benthic areas of cold-water corals and sponges in Atlantic Canada and the Eastern Canadian Arctic (see [45–46]), Beazley and colleagues [47] employed random forest modelling to predict the distribution of corals and sponges across DFO’s Maritimes Region administrative boundary. That work documented exploratory methods, including modelling with balanced and unbalanced response classes, biomass regressions and generalized additive models. Predictive maps were developed for *V*. *pourtalesi* separately from other sponges as there was confidence that *V*. *pourtalesi* catch would be reliably recorded at sea by non-experts given the unique morphology of the species.

Using more recent distribution data and higher-resolution environmental data, we here utilize random forest to predict the probability of occurrence and range distribution of *V*. *pourtalesi* across the Scotian Shelf. The physical oceanography of the Scotian Shelf and its basins has been well characterized in terms of water mass structure, circulation, and wind- and tidal-induced forcing [48–52]. However, the relationship between these patterns and the distribution of the *Vazella* sponge grounds has never been ascertained. We interpret the results of random forest modelling in light of the hydrographic conditions that govern Emerald Basin and the Scotian Shelf in order to make meaningful conclusions on the environmental conditions associated with these unique sponge grounds. We further examine historical oceanographic conditions to deduce any temporal patterns in the water masses surrounding this presumably long-lived species.

## Methods

### Study area and environmental setting

**Fig 2:**
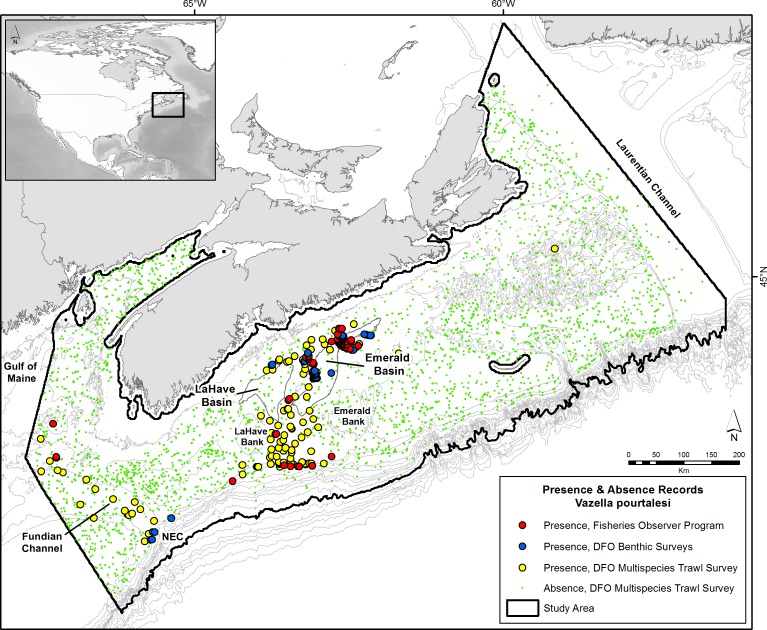
Distribution of *V*. *pourtalesi* on the Scotian Shelf and Gulf of Maine off Nova Scotia, Canada. Data are from the DFO multispecies trawl survey (yellow circles; 2007–2017), DFO benthic science surveys (blue circles; 2001–2017), and commercial bycatch records from the Fisheries Observer Program (red circles; 1997–2007, 2010–2015). Green circles are absence of *V*. *pourtalesi* from the DFO multispecies trawl survey. The 200-m contour defining Emerald and LaHave Basins is outlined in dark grey. Study area (black outline) is based on DFO’s Maritimes Region administrative boundary clipped to the 2000-m contour. NEC = Northeast Channel.

The Scotian Shelf (Fig 2) is a wide (up to ~200 km) and long (~700 km) continental margin off Nova Scotia, Canada that is characterized by a number of banks, deep basins, channels, and submarine canyons. It is separated from the Grand Banks in the east by the Laurentian Channel, and from the Gulf of Maine by the Northeast and Fundian Channels in the west. On the central Scotian Shelf lies an area commonly referred to as the ‘Scotian Gulf’, an inlet formed by a cross-shelf channel situated between Emerald and LaHave Banks which opens up into the LaHave and Emerald Basins on the inner shelf (Fig 2). Emerald Basin is composed of two basins. The main part (main basin) is 50 km wide, 100 km long, and nearly 300 m deep at its deepest point. The northern part (northern basin) is 10 km wide, 50 km long, and 230 m deep.

The Scotian Shelf lies at a confluence of two large ocean gyre currents: the subpolar Labrador Current and subtropical Gulf Stream [53]. The inner shelf is dominated by outflow from the Gulf of St. Lawrence and Labrador Current through Cabot Strait, which bifurcates to feed the Nova Scotia Current that moves westward along the inner shelf and a shelf-edge flow that moves along the western side of the Laurentian Channel and outer shelf [53]. On the outer shelf the Labrador Current has a more direct influence through flow across the Laurentian Channel from the southern Newfoundland Shelf. Offshore, warm North Atlantic Central Water sits below the cold shelf-edge flow and the warm and cold water masses variously mix to create a region of slope water with identifiable Labrador Slope Water (4–9°C) and Warm Slope Water (9–12°C) signatures [54]. This warm slope water moves onshore to fill the inner basins of the Scotian Shelf and the Gulf of Maine in the west. Intermittent incursions of Warm Slope Water through the Scotian Gulf result in relatively warm waters in the basins year round compared to the surrounding shelf, with temperatures typically in the range of ~8–10°C below ~100 m depth. The Warm Slope Water entering the basins is not only typically warmer and more saline, but similar to areas of upwelling at the shelf-break front it is also nutrient-rich relative to shelf water [55].

Emerald and LaHave Basins are also under the intermittent influence of two cold-water currents. The Nova Scotia Current is centred at about 150 m depth and flows over the northern part of Emerald Basin and bends southward to fill LaHave Basin. In addition, an onshore meander of the shelf-edge current flows northward over Western and Emerald Bank to join a weak anti-cyclonic gyre that flows around the inner-shelf basins [56]. Additionally, Emerald Basin is subjected to high tidal variability and mixing compared to other areas on the shelf [52]. The possible influence of this tidal mixing on the retention of particles and food in the basins has not been fully described. Azetsu-Scott and colleagues [57] described the presence of intermittent, intermediate nepheloid layers (i.e. layers of water that contains high amounts of suspended sediment) 10–30 m thick between 140 and 210 m depth in the main basin of Emerald Basin, approximately 90 m above the sea bed. Such nepheloid layers are often caused by biological patchiness in the water column, intrusion of turbid water, turbulence caused by bottom current stress, and/or by internal waves [57]. Azetsu-Scott and colleagues [57] suggested that these intermediate layers were caused by resuspension of particles by internal tides along the saddle between Emerald and LaHave Banks, and further transport by variable flows and the mean circulation of the Scotian Gulf. Resuspension by intrusion of slope water was thought to be important during the winter months when strong eastward winds and upwelling events cause strong, onshore flow and significant flushing of Emerald Basin [50]. This flushing is the likely cause of the disproportionate amount of the organic matter derived from marine, versus terrestrial sources, in Emerald Basin compared to other areas of the shelf [58].

The surficial geology and sediment stratigraphy of Emerald Basin and the Scotian Shelf has been well described based on acoustic techniques and bottom samples [59–61], from which broad-scale categorical maps have been generated [59]. Generally, the Scotian Shelf is comprised of five sediment formations (Scotian Shelf Drift, Sambro Sand, Emerald Silt, LaHave Clay, and Sable Island Sand and Gravel) that occur as an approximately 50 m-thick surficial succession over the bedrock [60,62]. Emerald Basin is overlain by Scotian Shelf Drift (i.e., glacial till), a hard substrate of variable size distribution that occurs in patches over a soft-sediment matrix. Based on *in situ* observations and physical collections, the presence of this hard substrate is crucial to the settlement of *V*. *pourtalesi*, which has been found attached to small pebbles dispersed over soft sediment up to boulder-sized substrate. In the Northeast Channel, *V*. *pourtalesi* is found on large rocks and boulders down to nearly 500 m depth where it co-occurs with large branching corals *Primnoa resedaeformis* and *Paragorgia arborea* [63].

### Spatial extent of study area

The study area used for data extraction and modelling (Fig 2) was adapted from the boundary of DFO’s Maritimes Region, one of DFO’s six administrative regions across Canada. This area encompasses the entire Scotian Shelf and is delimited by the 200 nautical mile Exclusive Economic Zone in the south, the Canadian Maritime Boundary to the west in the Gulf of Maine, and DFO’s Newfoundland and Labrador Region’s Placentia Bay–Grand Bank Large Ocean Management Area (LOMA) in the east. The boundary was clipped to the 2000 m isobath, as the deepest known record of *V*. *pourtalesi* was found < 1000 m depth (845 m off the Azores; [37]), the training data for the current model are <1850 m depth, and previous modelling applications of this species indicated that areas below 2000 m are considered extrapolated [47]. A 5 km buffer was placed around all land points to avoid their inclusion in the model.

### *Vazella pourtalesi* response data

*V*. *pourtalesi* presence and absence data (Fig 2 and S1 Table in supplementary material) were obtained from several different sources: DFO’s multispecies trawl survey conducted in the Maritimes Region between 2007 to 2017 (presences and absences), DFO optical (in-house camera/video and remotely operated vehicle) benthic surveys conducted between 2001 and 2017 (presences only), and commercial bycatch records from the Fisheries Observer Program (FOP) from 1997 to 2007, and 2010 to 2015 (presences only). The DFO multispecies trawl survey is stratified random (by depth) and conducted using primarily Western IIA trawl gear. The average distance of these tows is ~ 3.17 km. Absence records were created from null (zero) catches that occurred in the same surveys. Commercial bycatch data between 1997 to 2007 from the Fisheries Observer Program was further post-processed and validated for accuracy (see [35] for more details), while the data from 2010 to 2015 was extracted directly from the Maritimes Fishery Science Database managed by DFO. Commercial trawls are much longer in duration and may follow bottom contours and/or retrace their footprint through the course of a 10+ km tow. For both the DFO multispecies trawl survey and FOP data, start coordinates were used to represent the tow, whereas for the DFO benthic imagery survey data the actual location of the *V*. *pourtalesi* record *in situ* was used. A 1 x 1 km grid matching that of the environmental data was placed over the study area and the presence-absence data were reduced to one record per cell, with a presence taking precedence over an absence if both occurred in the same cell. This gave a total of 215 presences (102 from the DFO trawl surveys, 47 from DFO benthic science surveys, and 66 from the FOP) and 2867 absences for the model.

The inclusion of response data with different rates of detection or "catchability", spatial bias, and spatial resolution may introduce bias in species distribution models. For instance, the optical surveys used in this study were targeted towards areas of interest (i.e., areas of known sponge distribution), have a high rate of detection, low spatial coverage and high resolution, compared to the RV trawl survey and commercial bycatch data which aggregate the catch across larger areas (i.e., ~3 km and ~10+ km, respectively). Use of null data from the research vessel survey only will introduce another form of bias in terms of false negatives due to poor catchability, but as the absence data dominate the records and are generated from a random stratified survey, we consider any such biases to be negligible compared to those of the presence data. Ideally, models would be conducted with a single data type with random stratification across the study area (i.e. in this instance, using only those records from the RV trawl surveys). Given the scarcity of presences in the trawl survey data and differing spatial coverage from the optical surveys and commercial data, we chose to accept the biases incurred from combining all three datasets. We note however that the resulting predictive surfaces may be driven by such biases, particularly towards areas of aggregation of the optical survey and commercial bycatch data, which are not accompanied by absences records. Similarly, we caution against over-interpreting the boundaries of suitable and unsuitable habitat predicted by the model as a result of this spatial bias.

### Ethics statement

This study utilized data on the invertebrate sponge *V*. *pourtalesi* collected by other research programs or data sources. From those studies, data collected using optical techniques were non-destructive, while data collected by commercial and research trawls involved bringing the sponges on deck where they would have been exposed to air and died. Generally dead sponges collected in the trawls were returned to the sea near the point of sampling. Samples collected by others during the research vessel multispecies surveys conducted by Fisheries and Oceans Canada were done so with authorization to engage in fishing and related activities on the Atlantic coast of Canada subject to the provisions of the Fisheries Act and Regulations bestowed by the Regional Director of Science, Science Branch, Fisheries and Oceans Canada, Maritimes Region, Dartmouth, Nova Scotia, Canada. The field studies did not involve endangered or protected species.

### Environmental data

A total of 63 environmental data layers were considered as predictor variables in the random forest model. These variables are considered to represent current climate conditions (maximum temporal range is 1990 to 2015) and were derived from various data sources and native spatial resolutions. Variables were chosen based on their availability and assumed relevance to the distribution of *V*. *pourtalesi*. Biochemical variables such as oxygen, silicate, nitrate, and phosphate, some of which were used to model the glass sponge *Pheronema carpenteri* [64], were considered but rejected due to their poor spatial coverage in this region [65].

Hydrographic data (e.g. bottom and surface temperature, salinity, current velocity, and bottom shear) were extracted from a North Atlantic model termed BNAM (described in [66]) developed at the Bedford Institute of Oceanography in Dartmouth, Nova Scotia, Canada. BNAM is based on the NEMO (Nucleus for European Modelling of the Ocean) 2.3 reanalysis model, which includes both an ocean component (OPA, Océan PArallélisé; [67]) and a sea ice module (LIM, Louvain-la-Neuve Sea Ice Model; [68]). BNAM has a horizontal resolution of 1/12°, equating to ~ 6 km on the Scotian Shelf. Data from the period 1990 to 2015 were extracted.

Seasonal sea surface chlorophyll *a* from 2002 to 2012 and primary production data from 2006 to 2010 were also extracted. Chlorophyll *a* was derived from Aqua-MODIS (Moderate Resolution Imaging Spectroradiometer) Case I was processed by the Remote Sensing Unit at the Bedford Institute of Oceanography (RSU-BIO). Specific details on how these data were processed can be found in [65]. Annual and seasonal averages were computed, with seasons delimited by the following day of year ranges: days 91–181 (spring), 182–273 (summer), and 274–365 (fall). Seasonal (spring, summer, fall) and annual primary production layers were also used in the model, the details of which are summarized in [65].

Fishing effort from the time period of 2005–2014 from mobile bottom-tending gears was also included as a predictor in the model to ascertain whether fishing effort has shaped the distribution of *V*. *pourtalesi*. This predictor variable describes the sum of the fishing vessel monitoring system (VMS) ping time (in hours) within each 1 x 1 km grid cell, where a higher number of hours indicates higher effort, and was compiled for an analysis of the overlap between fishing effort and Significant Benthic Areas of cold-water corals and sponges in eastern Canada (see [69] for more details).

Emerald Basin is characterized by fluctuations in temperature and salinity that are higher between years than between seasons, and therefore we chose to use metrics of each oceanographic variable type that would describe both mean climate and the variability in oceanographic conditions across its temporal range. For each variable type (e.g., bottom temperature), four different statistical quantities were calculated across its temporal data range: minimum, maximum, mean and range (difference between minimum and maximum). This was done by averaging the minimum, maximum, or mean values between all years of the dataset. All variables except depth and slope were then spatially interpolated across the study area using ordinary kriging in ArcMap 10.2.2 software [70] to create continuous data surfaces with a ~1 km grid size. All predictor layers were displayed in raster format with geographic coordinates using a WGS 1984 datum and a ~0.012° cell size (approximately equal to 1 km in our study region). Slope was derived from depth projected using the NAD 1983 Zone 20N coordinate system (units in metres), and both depth and slope were re-projected using a WGS 1984 datum and resampled to the 0.012° grid size using the nearest neighbour technique. Averaged bottom temperature and salinity from the BNAM model were additionally used to illustrate the association of *V*. *pourtalesi* with regional water masses.

Broad-scale categorical maps of the surficial geology of the region have been generated [59], and as noted above, *V*. *pourtalesi* attaches to hard substrate. Such categorical data were not included as predictors in our model for several reasons: 1) they did not fully cover the known distribution of *V*. *pourtalesi* on the Scotian Shelf, 2) random forest has shown bias towards categorical variables with a high number of levels [71], and 3) patterns in habitat heterogeneity on the Scotian Shelf occur over micro-scales [72–73]; therefore such data would not be useful in explaining patterns in the spatial distribution of *V*. *pourtalesi* with the resolution of our model.

Although the performance of classification random forest models are relatively unaffected by the presence of correlated predictor variables [74], correlated variables may have a significant effect on variable importance measures and ecological interpretation, showing bias towards the most highly correlated predictors [75]. As most of the variables were moderately or highly correlated, only very strongly correlated variables (Spearman’s rank correlation coefficient *ρ* ≥ 0.9; [76]) were eliminated. Variable elimination was done following the procedure outlined by [77]. Spearman’s rank correlation coefficients were calculated between predictor variables for each raster grid cell in the study area. The two predictors with the highest correlation were considered and one eliminated based on user-defined criteria. In these criteria, those variables considered more important to the distribution of deep-water sponges (e.g., near-bottom processes over surface) were favoured, as well as those variables considered to have a more direct (e.g. bottom temperature) than proximal (e.g. depth) influence on species’ distributions. The process was repeated until no variables correlated > 0.9 remained. Table 1 shows the remaining 35 variables used in the random forest model. A depiction of the Spearman’s rank correlation between the 63 original variables used to make this selection can be found in the supplementary material (S1 Fig).

**Table 1:** Environmental predictor variables remaining after variable elimination and included in the random forest model. NA = not applicable.

Variable	Metric	Unit	Native Resolution
Bottom Temperature	Min, Max, Range	°C	1/12^th^ degree
Bottom Salinity	Min	NA	1/12^th^ degree
Bottom Current	Max, Min	m s^-1^	1/12^th^ degree
Surface Temperature	Max, Range	°C	1/12^th^ degree
Surface Salinity	Range	NA	1/12^th^ degree
Surface Current	Min, Max	m s^-1^	1/12^th^ degree
Annual Surface Chlorophyll *a*	Max, Range	mg m^-3^	2 km
Spring Surface Chlorophyll *a*	Min, Max, Mean	mg m^-3^	2 km
Fall Surface Chlorophyll *a*	Min, Max	mg m^-3^	2 km
Annual Surface Primary Production	Min, Max, Mean, Range	mg C m^-2^ day^-1^	9 km
Spring Surface Primary Production	Min, Max, Mean, Range	mg C m^-2^ day^-1^	9 km
Summer Surface Primary Production	Min, Max, Range	mg C m^-2^ day^-1^	9 km
Fall Surface Primary Production	Min, Max, Mean, Range	mg C m^-2^ day^-1^	9 km
Bottom Fishing Effort	NA	Percentages	1 km
Slope	NA	Degrees	15 arc-sec

### Random forest modelling

Random forest classification [78] was used to predict the probability of occurrence and range distribution of *V*. *pourtalesi* based on the 215 presence and 2867 absence records and 35 predictor variables as described above (Table 1). Random forest is a non-parametric machine learning technique where multiple trees are built using random subsets of the response data. Each tree is fit to a bootstrap sample of these data, and the best predictor at each node is that which splits the response data so that maximum homogeneity is reached in the child nodes. Models were fitted using the ‘randomForest’ package [79] in the statistical computing software program R version 3.3.1 [80]. Default values were used for RF parameters, and the default 500 trees were constructed.

Model performance was assessed in R using 10-fold cross validation. In this process the response data are split into 10 folds of equal size, and a model is trained on a combination of 9 folds and validated on the remaining fold. The process is repeated 10 times, giving 10 repetitions for which accuracy measures are derived. Sensitivity (i.e., the proportion of accurately predicted presences) and specificity (the proportion of accurately predicted absences) were derived by summing the predicted outcomes across the 2 x 2 confusion matrices generated for each of the 10 model folds. Low sensitivity represents high omission error (i.e., false negative rate), while low specificity indicates high commission error (i.e., false positive rate). The response dataset for *V*. *pourtalesi* is characterized by a higher number of absences relative to presences (i.e., unbalanced species prevalence, where prevalence is the proportion of presences in relation to the total dataset). Classification accuracy in random forest is prone to bias when the categorical response variable is highly imbalanced [81]. This is due to over-representation of the majority class in the bootstrap sample leading to a higher frequency in which the majority class is drawn, therefore skewing predictions in that favour [82]. Given the highly imbalanced nature of the response dataset, a threshold defining above which a class probability is considered a presence is often used to convert the class probabilities into predicted outcomes that are then summarized in the 2 x 2 confusion matrix. We used prevalence, or the probability of presences in the training dataset, as the threshold defining when a species is considered present [83–84]. The discrimination capacity of the training model was determined by calculating the average Area under the Receiver Operating Characteristic (ROC) Curve, or AUC, across all 10 model folds. The AUC is considered threshold-independent and is calculated from a combination of the true positive rate and false positive rate (1-specificity). AUC equals the probability that the model will rank a randomly-chosen presence instance higher than a randomly-chosen absence instance [85], where values > 0.9 indicate excellent model performance, 0.8–0.9 very good, 0.7–0.8 good, 0.6–0.7 fair, 0.6–0.5 poor, and <0.5 no better than random.

Using R’s ‘predict’ function, a model trained on all of the data (215 presences and 2867 absences) was used to predict the probability of presence of *V*. *pourtalesi* across the entire study area, creating a 1 x 1 km raster grid surface of predicted presence probabilities. Additionally, the probabilistic map was converted into a discrete map showing areas of suitable and unsuitable habitat using the prevalence threshold, where cells with probabilities less than the threshold were considered unsuitable habitat, and those greater considered suitable. The true skill statistic (TSS), which maximizes the sum of sensitivity and specificity and is considered to have all the advantages of the kappa statistic but is independent of prevalence [86], was also considered in this study. It was very similar to the prevalence threshold (0.11 versus 0.07 for prevalence) and resulted in only a slightly reduced area predicted as suitable habitat. For *V*. *pourtalesi*, a long-lived species vulnerable to fishing impacts, our goal was to reduce the omission error (where the model predicts an absence where a presence is located) as much as possible and therefore we applied the prevalence threshold in preference to the TSS.

Ecological context of the model was aided by predictor variable importance and response curves (partial dependence plots). The importance of the predictor variables in the classification model was assessed using the ‘importance’ function of package ‘randomForest’, which calculates the Mean Decrease in Gini index, or Gini impurity for each variable. When the response data are split into two child nodes based on a randomly-chosen variable, the data in the two descendent nodes are more homogeneous than that of the parent node. This difference in homogeneity between parent and child nodes is measured by the Gini index, where the increase in homogeneity equals a decrease in Gini value. The sum of all decreases in Gini index for each variable in each tree is averaged across all trees in the model. The variable with the highest mean decrease in Gini value is considered the most important variable in the model.

Response curves showing the partial dependence on the top six predictor variables were generated using the ‘partialPlot’ function in R. For classification random forest, these partial dependence plots show the marginal effect of a predictor variable on the class probability. The other predictor variables are held constant at their mean observed value. Partial dependence plots are useful in showing general trends in model accuracy’s dependence on the predictors [87]. For classification models, the *y* axis ranges from -∞ to ∞ and quantifies the log-odds of a positive classification for the total range of values in *x*. Log-odds are logarithmic transformations of the probabilities for values in *x* [88]. These values were transformed to the original presence probability scale using: *p* = *exp*(*y*)/(1 + *exp*(*y*)), where p = the probability of presence, and y is the log-odds of presence, the standard output from the partialPlot function.

### Fine-scale hydrographic conditions over the *Vazella* sponge grounds

Fine-scale hydrographic and biochemical conditions directly over the *Vazella* sponge grounds were examined from four CTD profiles made on July 20, 2016 as part of a DFO science mission to characterize the environmental characteristics of these sponge grounds (see [89]). Four continuous, full-depth profiles of temperature, salinity, fluorescence, density, and oxygen were made using a CTD and rosette with 24 10-L sampling bottles to collect water samples for the analysis following standard procedures [90] of silicate, nitrate, and phosphate through the water column to 10 m above the seabed. Note that nutrient data was only acquired for three of the four casts. CTD casts were made in the northeast corner of DFO’s Emerald Basin *Vazella* Conservation Area in a gradient from high sponge densities to low. CTD casts were approximately 1 km apart.

### Climatological trends and inter-annual variability in water mass characteristics over the *Vazella* sponge grounds

Long-term trends in bottom temperature and salinity in Emerald Basin were examined from two different sources: observational data from CTDs and ARGO floats collected in Emerald Basin between 1950 and 2015, and the Simple Ocean Data Assimilation (SODA) version 2.2.4 reanalysis model. The goal of SODA is to reconstruct the historical physical history of the global ocean through the optimization of model physics, forcing, and available ocean data observations. Gridded monthly data were extracted from SODA for the time period 1871 to 2010 from a bounding box with a longitude of -63.5W, -63W by latitude of 43.5N, 44.0N. As the first record of *V*. *pourtalesi* from the region dates back to 1889 [34], this timeframe can be used to describe the variability in conditions experienced by the *V*. *pourtalesi* population on the Scotian Shelf. Trends in temperature and salinity from SODA were displayed by season (winter: Jan. to Mar., spring: Apr. to June, summer: July to Sept., fall: Oct. to Dec.) and as annual averages.

## Results

**Fig 3:**
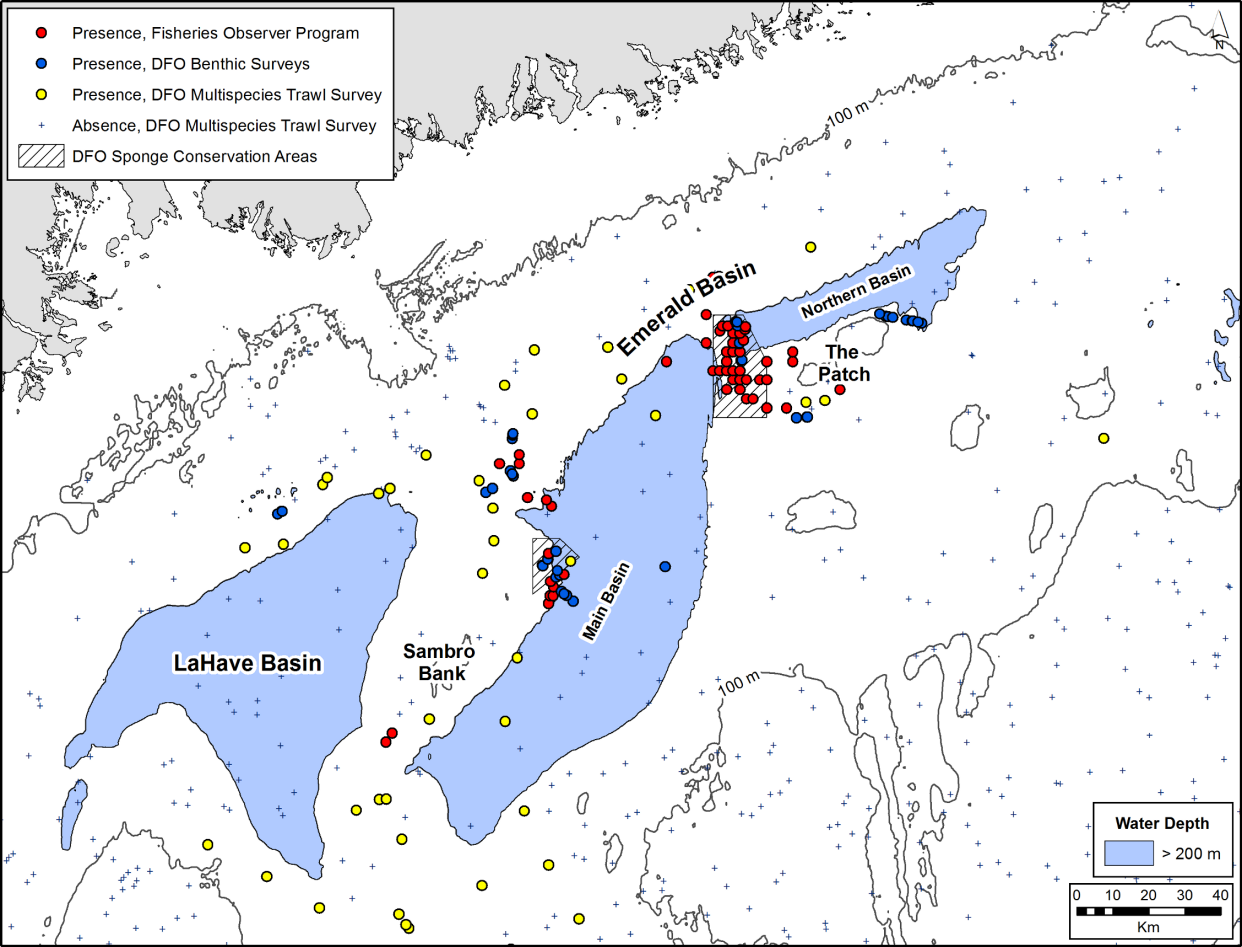
Distribution of *V*. *pourtalesi* from the commercial Fisheries Observer Program and DFO benthic imagery and multispecies trawl surveys in Emerald and LaHave Basins. Water depths greater than 200 m based on CHS bathymetry are shown in blue. The DFO Sponge Conservation Areas to protect *V*. *pourtalesi* are indicated by thatched boxes.

Observational data of *V*. *pourtalesi* indicated that this species aggregates on the flanks of the two deep-water basins that comprise Emerald Basin, with the densest concentration located between the main and northern basins and to the west of the area known to fisherman as ‘The Patch’ (Fig 3). This concentration is mostly captured by DFO’s Emerald Basin *Vazella* Conservation Area. A second notable concentration was situated on the southwestern portion of the main basin adjacent to Sambro Bank, and is partially protected by DFO’s Sambro Bank *Vazella* Conservation Area. *V*. *pourtalesi* was found in lower concentrations along the saddle between Emerald and LaHave Banks in the Scotian Gulf, and in the Northeast and Fundian Channels leading to the Gulf of Maine. *V*. *pourtalesi* was generally absent from the shelf, Bay of Fundy, and in off-shelf waters to ~1850 m depth. The overall depth range of presence observations was 87 to 498 m (based on Canadian Hydrographic Service Atlantic Bathymetry Compilation, 500-m bathymetry). The deepest records were found along the shelf break at the mouth of the Scotian Gulf and in the Northeast Channel. The shallowest record of this species was from Misaine Bank at 87 m depth. While some records are associated with high-slope areas (11° in the Northeast Channel), the densest sponge grounds occurred in areas of low topographic relief (0.04 to 3.20° in Emerald Basin).

### Predicted distribution of *Vazella pourtalesi* from random forest modelling

The random forest model predicting the presence probability of *V*. *pourtalesi* had an excellent performance rating, with a 10-fold cross-validated AUC of 0.967 ± 0.020 (SD). The sensitivity and specificity of this model were also high, at 0.940 and 0.889, respectively. These accuracy measures and the 2 x 2 confusion matrix are presented in Table 2.

**Table 2:** Accuracy measures and confusion matrix from 10-fold cross validation of a random forest model of presence and absence of the glass sponge *V*. *pourtalesi*. Observ. = Observations; Sensit. = Sensitivity, Specif. = Specificity. AUC represents an average across all 10 model folds, while the confusion matrix, class error, sensitivity and specificity result from a summation of the predicted outcomes in the 2 x 2 confusion matrix generated for each of the 10 model folds. Prevalence (0.07) was used as the probability threshold.

AUC	Observ.	Predictions		Total n	Class error	Sensit.	Specif.
		Absence	Presence				
0.967 ± 0.020	**Absence**	2550	317	2867	0.111	0.940	0.889
	**Presence**	13	202	215	0.060		

**Fig 4:**
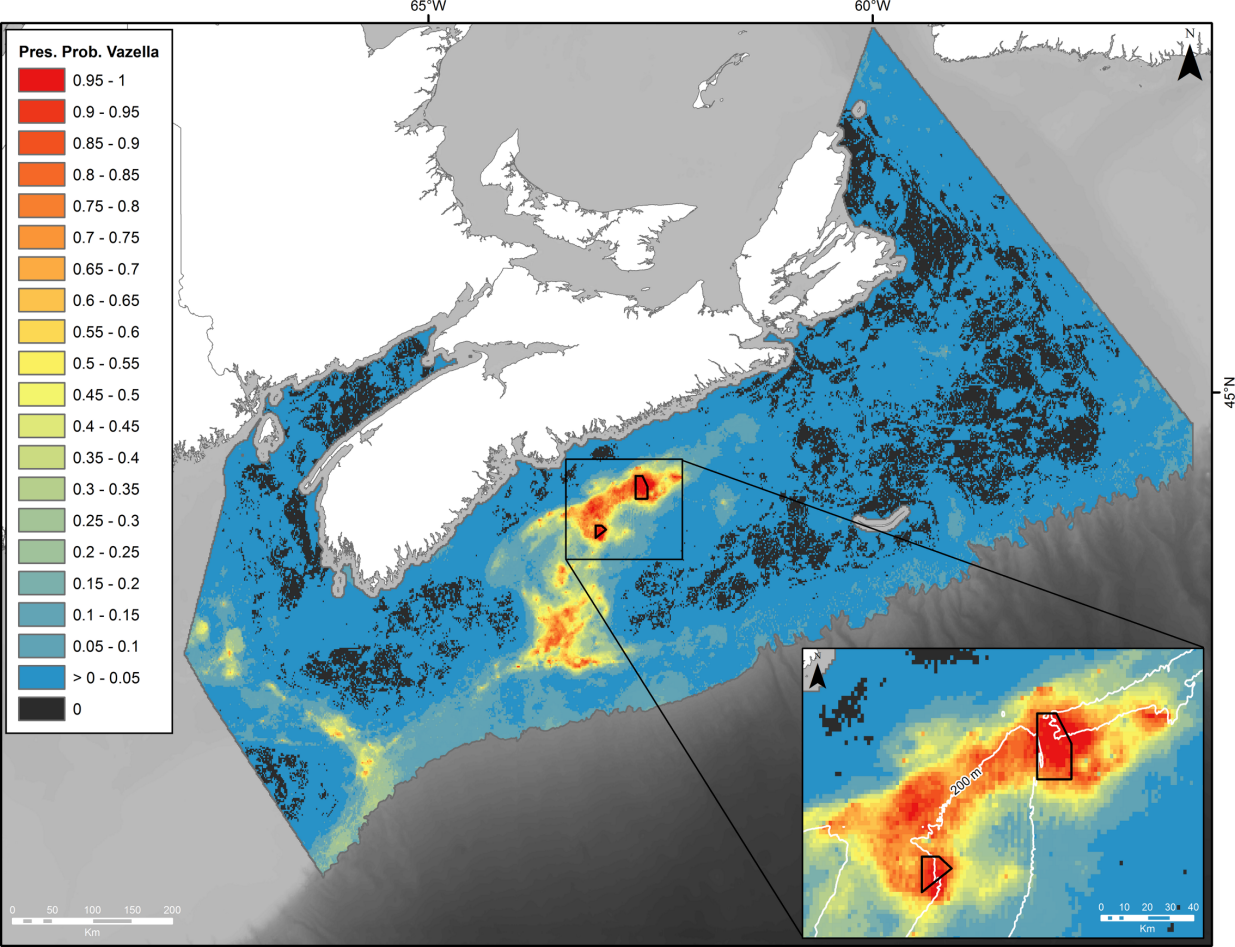
Predicted presence probability of *V*. *pourtalesi* from a random forest model built on *V*. *pourtalesi* presence-absence data. Boundary is based on DFO’s Maritimes Region administrative boundary clipped to the 2000-m depth contour. Inset shows the two DFO Sponge Conservation Areas and 200 m contour.

**Fig 5:**
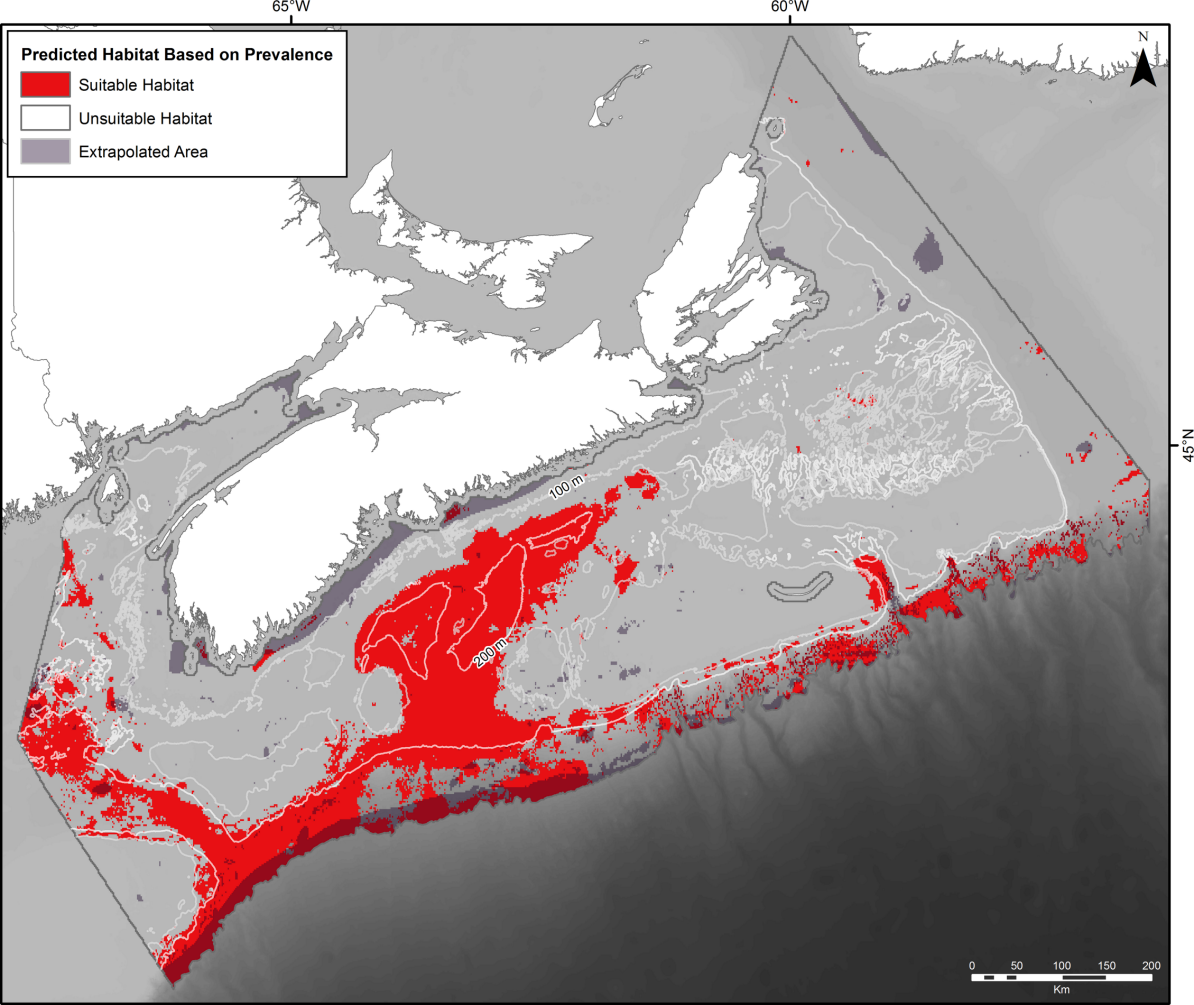
Predicted distribution of suitable (red) and unsuitable (blank) habitat for *V*. *pourtalesi* based on the prevalence threshold of 0.07 applied to the predicted probabilities from random forest. Also shown are areas of model extrapolation (grey, semi-transparent polygons) by the random forest model (i.e., those areas where the environmental data are outside the bounds of those used to train the model).

The probabilistic map from the random forest model indicated high presence probability of *V*. *pourtalesi* along a band in the northern portion of Emerald Basin (Fig 4). The highest probability was found to the west of The Patch where DFO’s Emerald Basin *Vazella* Conservation Area is situated, which corresponds to the location of the densest concentration of presence records (Fig 3 and S2 Fig). Lower presence probability was predicted in the deeper waters of LaHave and the main basin of Emerald Basin, where water depths are greater than 200 m (Fig 3). Generally, the surrounding shelf was predicted with either zero or low probability of occurrence. Smaller patches of high presence probability were found on the saddle between Emerald and LaHave Banks, and in the Northeast and Fundian Channels where sparse presence data are distributed. The shelf break to 2000 m in the western portion of the study area was predicted with moderate presence probability; however this area is considered extrapolated (i.e., outside the environmental envelope used to train the model) and thus the predictions here should be discounted (S2 Fig). Apart from the deepest areas of LaHave Basin, the entire Scotian Gulf was predicted as potential suitable habitat for *V*. *pourtalesi* once the probabilistic map was converted into discrete predictions of suitable versus unsuitable habitat using the prevalence threshold (0.07; Fig 5). A corridor of suitable habitat connected the Scotian Gulf and the Northeast and Fundian Channels.

**Fig 6:**
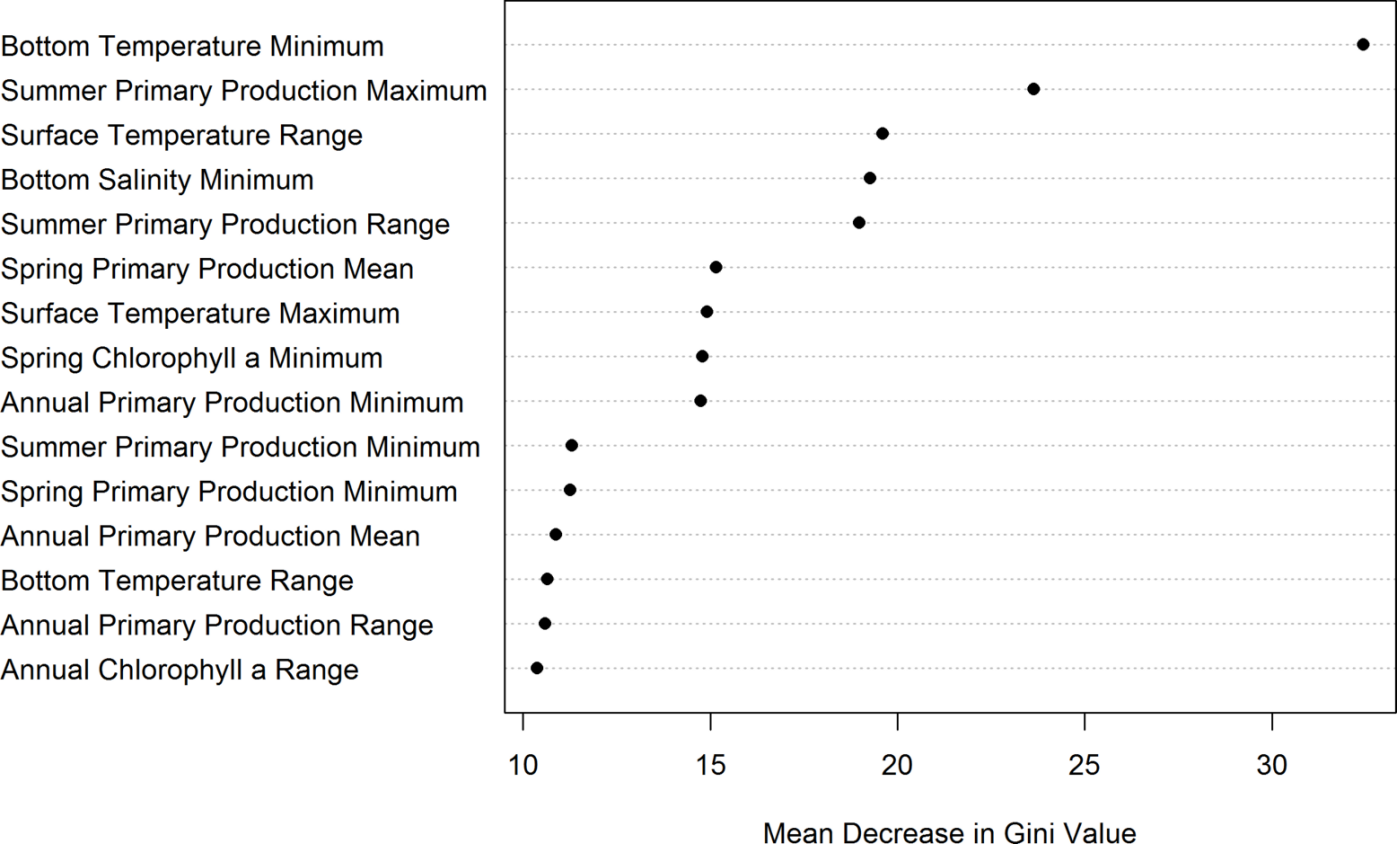
Mean decrease in Gini plot indicting the importance of each variable in the random forest model.

Bottom Temperature Minimum (i.e. the minimum bottom temperature per year at each location, averaged over the time series) was the most important predictor of *V*. *pourtalesi* presence probability in our model (Fig 6). This variable was most highly correlated with Bottom Temperature Mean (i.e. the average bottom temperature over the time series; Spearman’s rank correlation coefficient = 0.90), which was excluded during the variable elimination process. Bottom Temperature Minimum was followed more distantly in terms of the mean decrease in Gini index by Summer Primary Production Maximum (i.e., the maximum bottom temperature per year at each location, averaged over the time series) and Surface Temperature Range (where range equals the difference between maximum and minimum values per year, averaged over the time series; a measure of variability). Generally, variables related to primary production held a high importance in the model. Bottom fishing effort was the least important variable in the model.

**Fig 7:**
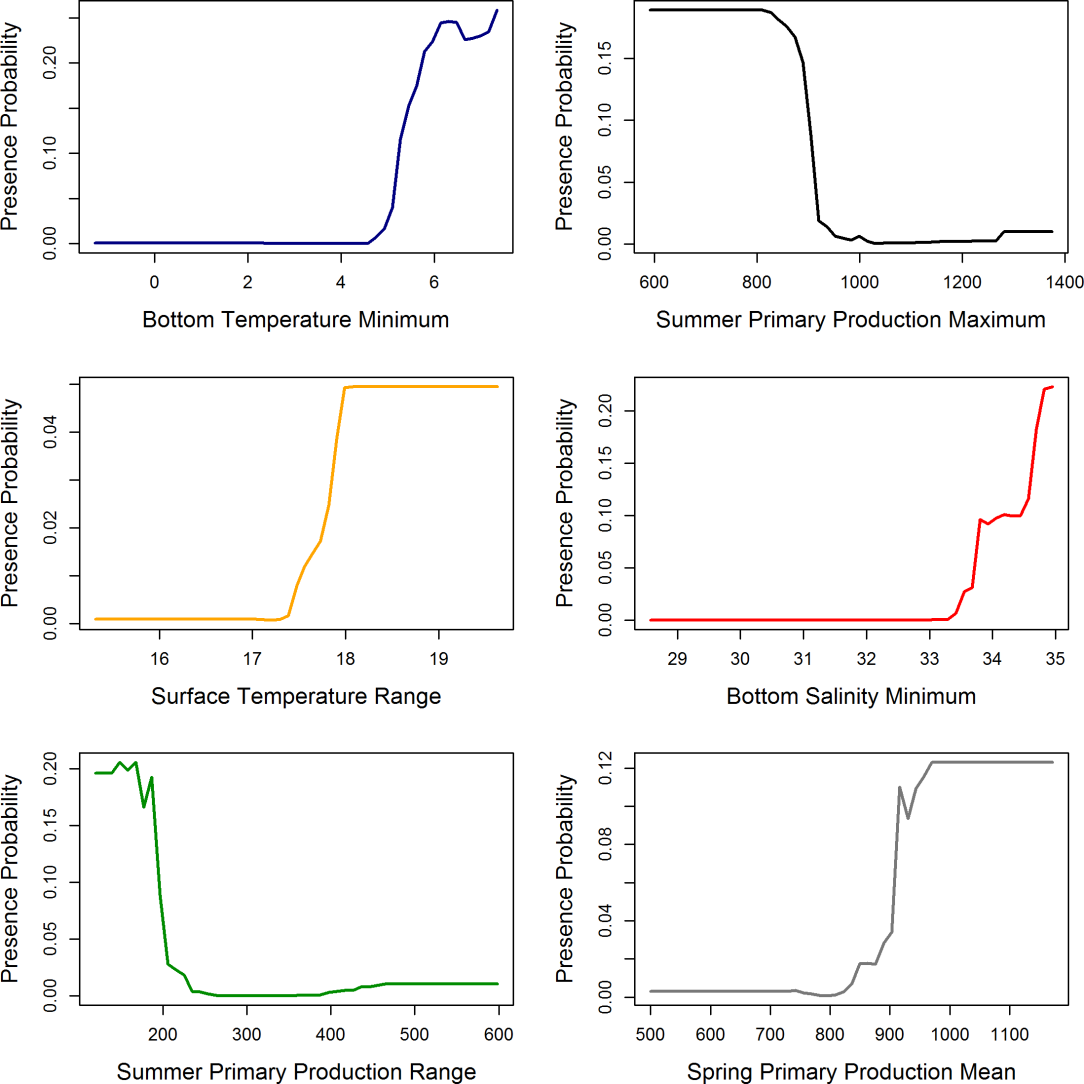
Response curves showing the partial dependence on the top (left to right, top to bottom) 6 predictor variables identified in the random forest model.

**Fig 8:**
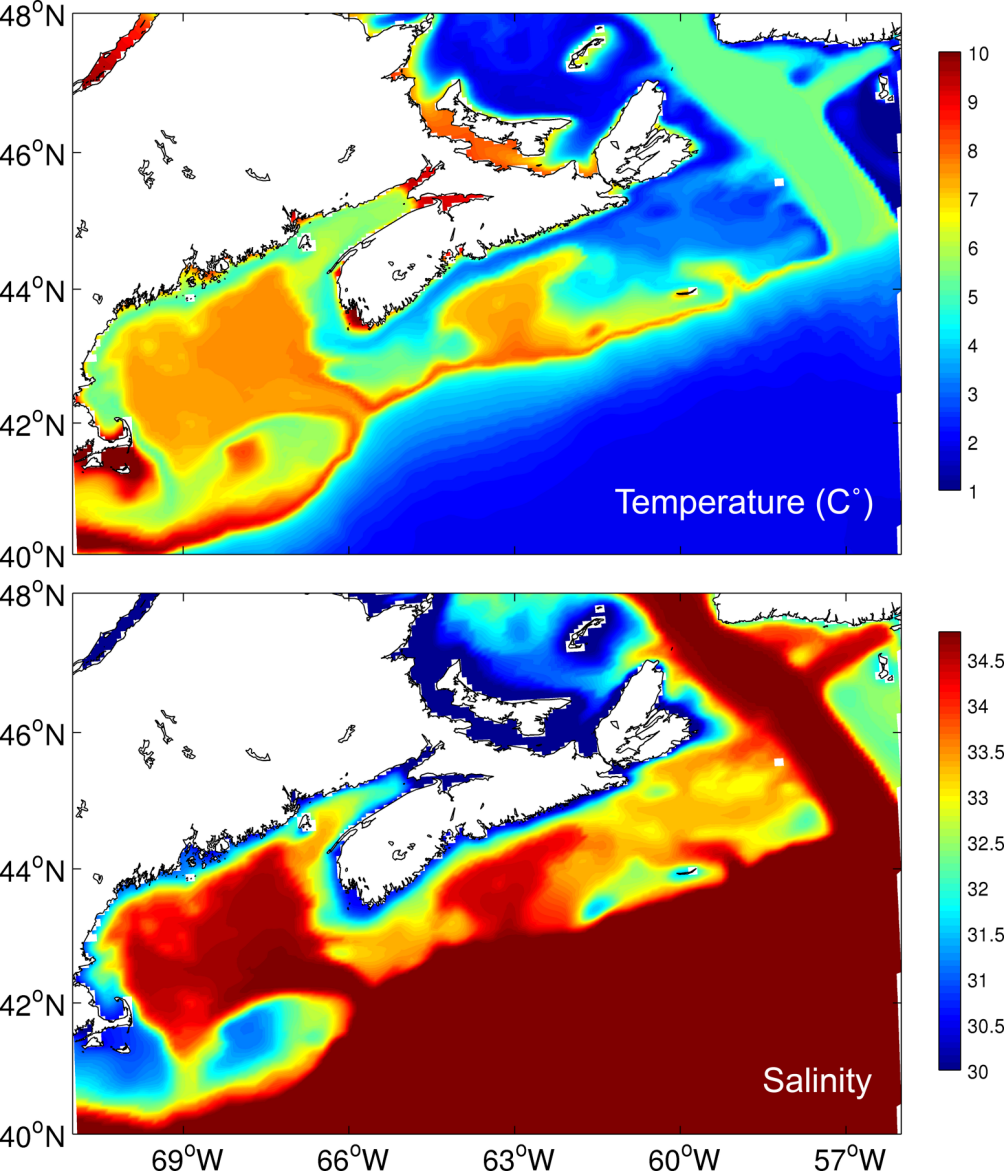
Mean bottom temperature (top panel) and salinity (bottom panel) from 1990 to 2015 extracted from BNAM.

The response curves of the top 6 predictor variables are shown in Fig 7. Higher presence probability of *V*. *pourtalesi* was associated with minimum bottom temperatures of 5°C and warmer, and bottom salinities of 34 and higher. The importance of bottom temperature and salinity in predicting the distribution of *V*. *pourtalesi* is further reflected through examination of the near-bottom temperature and salinity from BNAM (Fig 8). The predicted distribution of *V*. *pourtalesi* (Figs 4 and 5) followed closely the distribution of a warm and saline water mass that infiltrates the inner shelf basins through the saddle of the Scotian Gulf and the Gulf of Maine. This water mass follows the slope across the region where suitable habitat of *V*. *pourtalesi* was predicted in patches (Fig 5), but where no presences were recorded.

**Fig 9:**
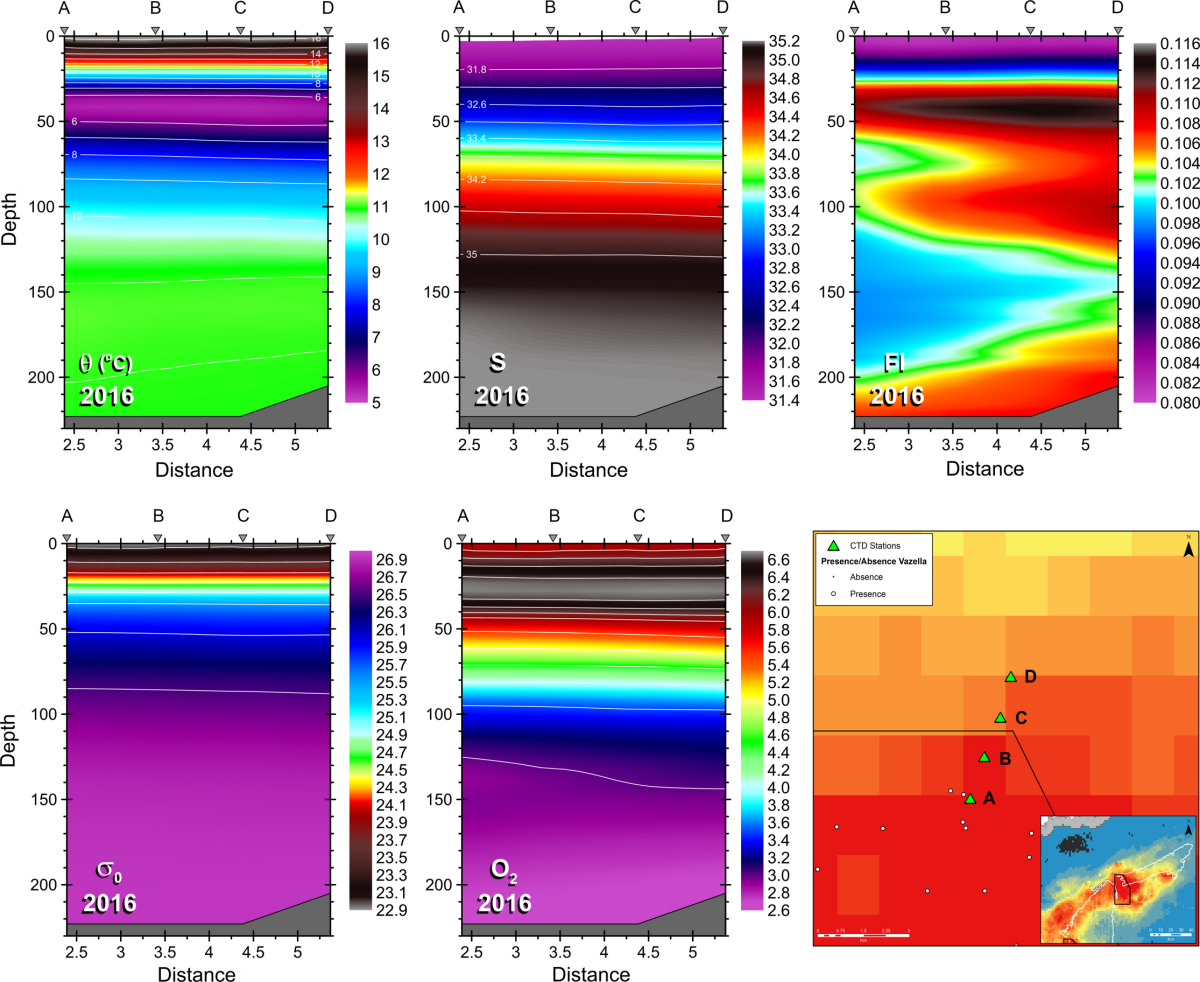
Temperature°C (θ), salinity (S), fluorescence (Fl), density (σ_0_) and oxygen (O_2_) profiles from four CTD casts collected on July 20, 2016 in DFO’s Emerald Basin *Vazella* Conservation Area, in a gradient across the predicted presence probability of *V*. *pourtalesi* from random forest (lower right panel). The y and x axes represent depth (m) and distance (km), respectively. The seabed is indicated by the grey area at the bottom of each profile. CTD casts are indicated at the top of each plot by the grey triangles and letters, which correspond to the locations of the casts (green triangles) in the lower right map. Nutrients were available from casts A, B, and D only. The coloured background map indicates the predicted presence probability of *V*. *pourtalesi* from random forest.

**Fig 10:**
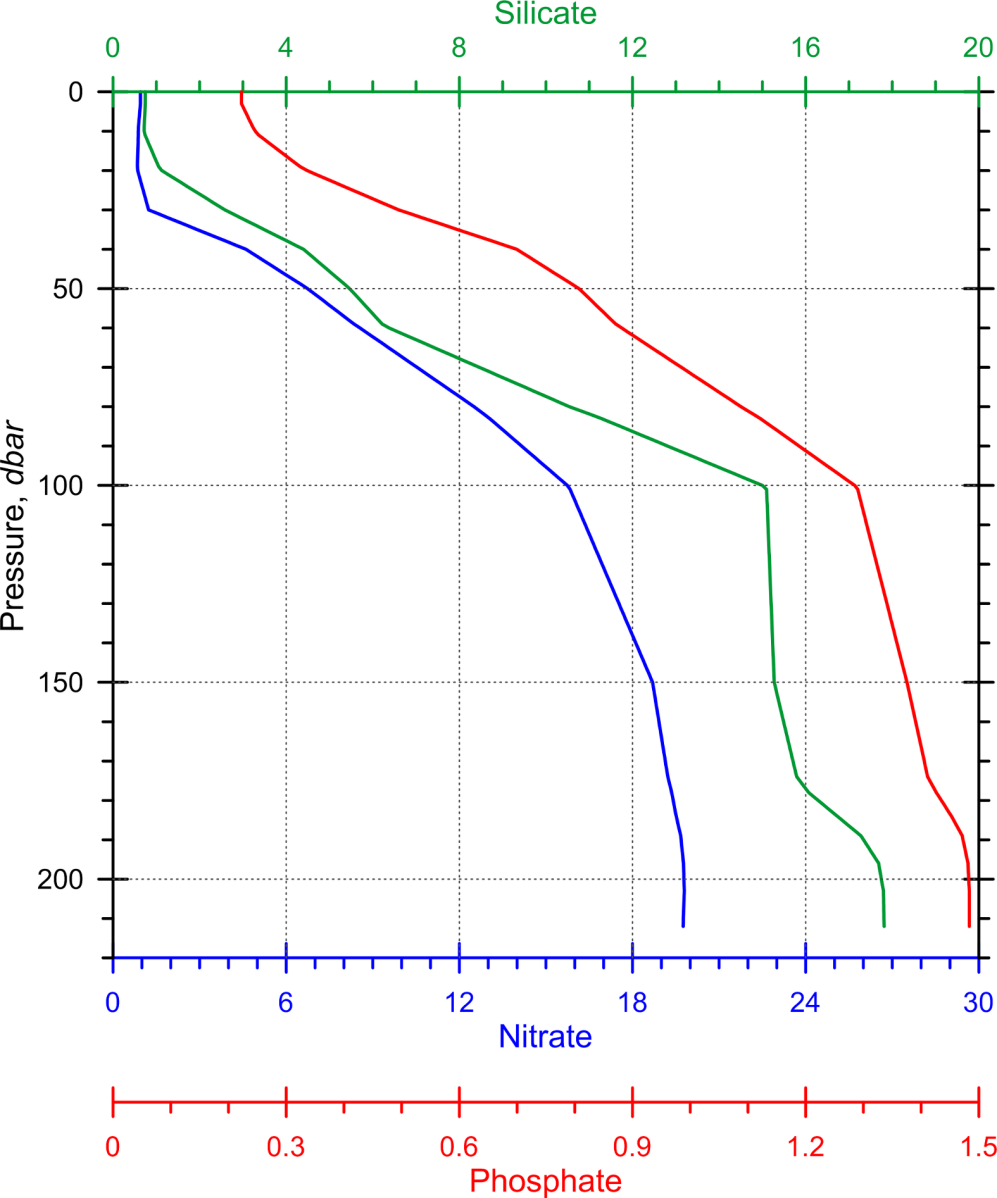
Mean concentration (mmol m^-3^) of silicate, nitrate, and phosphate averaged across three CTD casts (casts A, B, and D in Fig 9) collected over the *Vazella* sponge grounds in Emerald Basin in 2016. Continuous profiles are created by linear interpolation between sample depths.

Recent oceanographic data collected within the *Vazella* sponge grounds also confirms the presence of a warm, saline water mass over the bottom. Fig 9 shows profiles of temperature (°C), salinity, fluorescence (mg m^-3^), density (kg m^-3^), and oxygen (ml l^-1^) collected in Emerald Basin in 2016. The profiles indicate a warm (~11°C) and saline (>34) water mass above the sponge grounds that underlies colder and less saline water (Fig 9). This bottom water mass is denser in waters below 100 m depth, and low in oxygen directly above the sponge grounds. Fluorescence was higher above the sponge grounds than in the surface layers. Fig 10 shows the concentration of silicate, nitrate, and phosphate averaged across three of the four CTD profiles collected in Emerald Basin in 2016 (locations A, B, and D in Fig 9). The concentration of nutrients increased with depth but was relatively uniform within the deepest 100 m. Nitrate, phosphate, silicate, and ammonia (not shown in Fig 9) showed prominent peaks within 20 m from the seabed (Table 3). No notable differences that could be attributed to sponge presence were observed in the oceanographic (e.g., temperature and salinity) and biochemical (e.g., nutrients) parameters between the three CTD casts and across the gradient of high to medium predicted presence probability of *V*. *pourtalesi* (see Fig 9). This may be due to the scale of the resolution of the three CTD casts which were 1 km apart.

**Table 3:** Mean concentration ± SD (mmol m^-3^) of nitrate, nitrite, phosphate, silicate, and ammonia within 20 m from the seabed, and 20–40 m from the seabed from three CTD casts (casts A, B, and D in Fig 9) collected in DFO’s Emerald Basin *Vazella* Conservation Area (see Fig 9). The difference in nutrient concentration between these layers is also shown.

	Concentration (mmol m^-3^)		
Nutrient	Bottom 20 m layer	20–40 m layer	Difference
Nitrate	19.55 ± 0.55	19.20 ± 0.67	0.35 ± 0.18
Nitrite	0.20 ± 0.00	0.20 ± 0.00	0.00 ± 0.00
Phosphate	1.46 ± 0.02	1.42 ± 0.02	0.04 ± 0.00
Silicate	17.18 ± 0.70	15.96 ± 0.46	1.22 ± 0.36
Ammonia	0.53 ± 0.06	0.47 ± 0.04	0.05 ± 0.03

### Climatological trends and inter-annual variability in water mass characteristics over the *Vazella* sponge grounds

**Fig 11:**
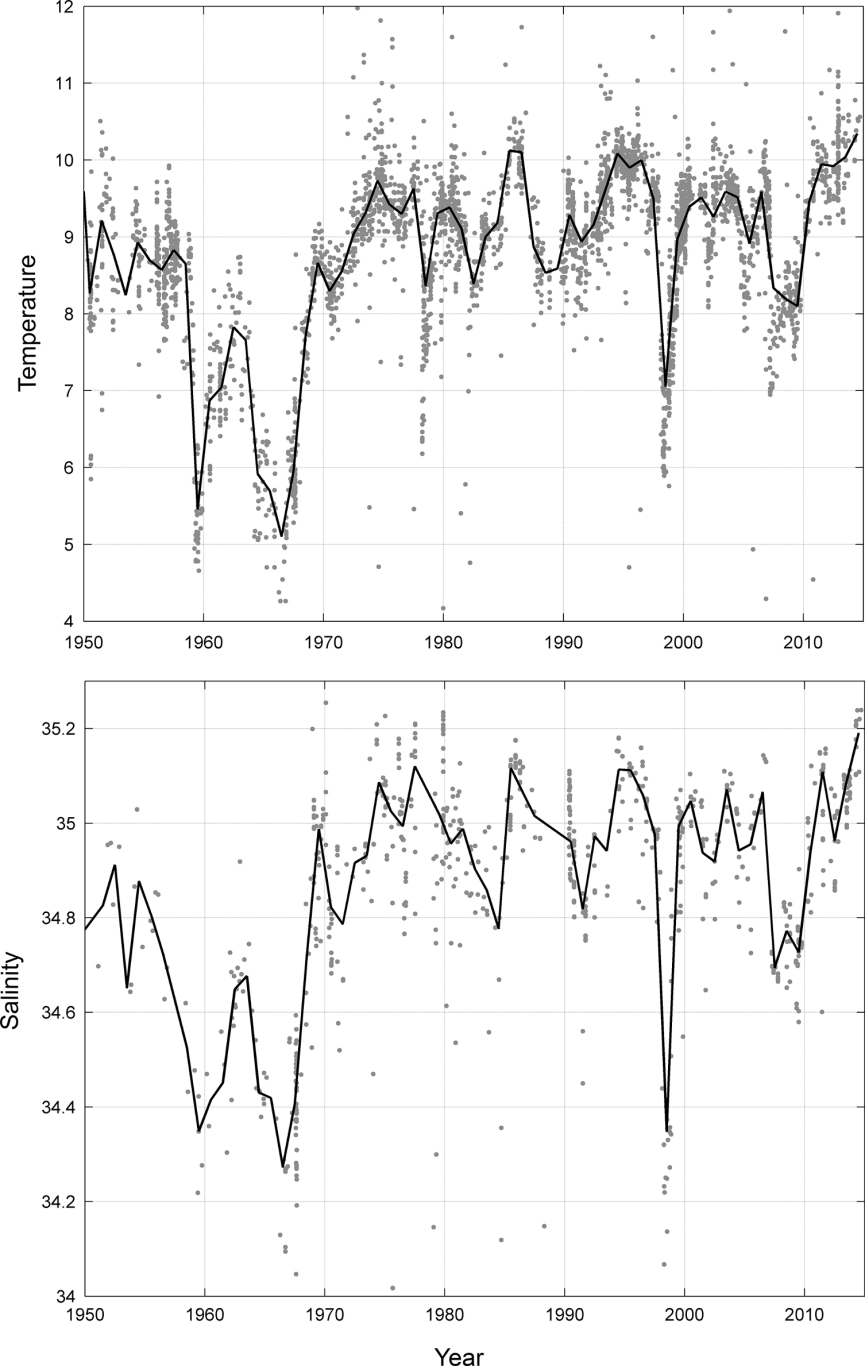
Near-bottom temperature (°C; top panel) and salinity (bottom panel) from 1950 to 2015 collected from CTD casts and ARGO float data collected from Emerald Basin. Trend line is shown in black.

**Fig 12:**
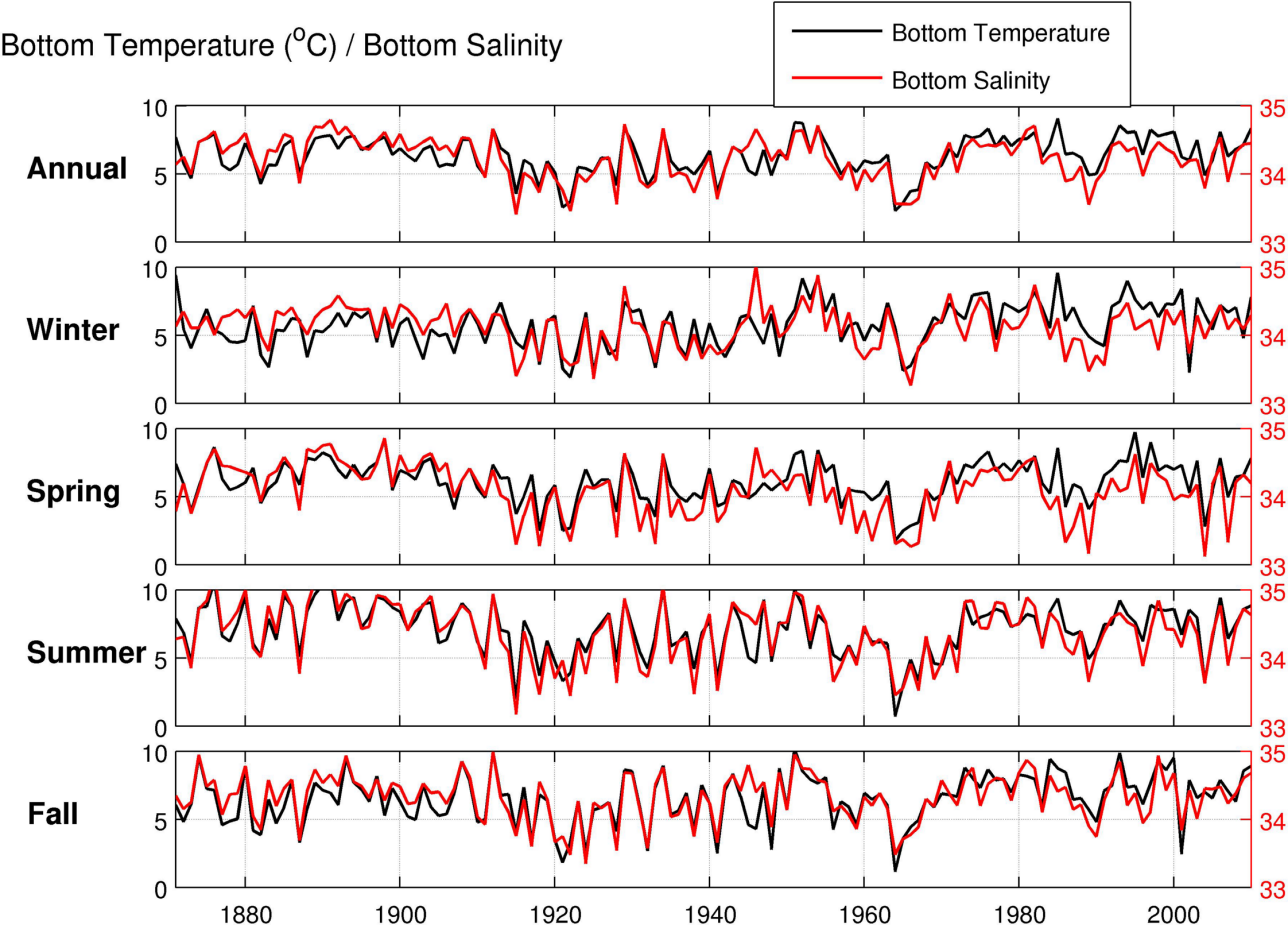
Annual and seasonal mean bottom temperature (°C; black lines) and salinity (red lines) in Emerald Basin between 1871 and 2010 from SODA.

Examination of both historical observational and modelled temperature and salinity data from the SODA model in Emerald Basin indicated that this area has experienced high inter-annual variability in these water mass characteristics. Fig 11 shows the near-bottom temperature and salinity from CTD casts and ARGO float data collected between 1950 and 2015 from Emerald Basin. The temperatures experienced over this time period ranged from 4°C in the mid 1960’s to upwards of 12°C. A second cold period was experienced in the late 1990's. Salinity also followed the same pattern, with fresher waters associated with the cold water mass experienced in the mid 1960’s and late 1990’s. The cooler/fresher waters were observed at the start of the time series with a general increasing trend in temperature/salinity to present day. The longer time series of temperature and salinity generated by the SODA model (Fig 12) also showed strong inter-annual variability in the water masses in Emerald Basin. The cold/fresh water mass of the 1960’s as shown in the observational data was reproduced here, and a second cold period in the 1920’s was also indicated. Limited seasonality in near-bottom temperature and salinity was also shown.

## Discussion

Our study represents the first description in the primary literature of a monospecific sponge ground formed by the glass sponge *V*. *pourtalesi* in Emerald Basin, Nova Scotia, Canada. The *Vazella* sponge grounds in Emerald Basin are globally unique for this species, which to date has not been reported to form large aggregations in other areas of its distribution, and are also unique for the northwest Atlantic where ostur-type sponges are typically the dominant sponge-ground type [28,77,91–92].

In response to the growing concern over the conservation status of deep-water sponges and the impact of anthropogenic activities such as bottom fishing to these communities [1], several studies have used species distribution modelling to predict the distribution of deep-water sponges and identify the environmental conditions responsible for their formation and persistence at both regional (e.g., [77,91,93–95]) and basin-wide (e.g., [64]) scales. In our study, predicted probability of occurrence of *V*. *pourtalesi* was highest in Emerald Basin in waters shallower than 200 m depth, with a significant concentration located to the west of ‘The Patch’. A second notable concentration was predicted on the eastern flank of Sambro Bank. Although a threshold delimiting significant concentrations of *V*. *pourtalesi* from its broader distribution from presence probability outputs remains undefined, the two DFO Sponge Conservation Areas almost fully encompass these concentrations, where presence probability was predicted at 90% and higher (see Fig 4). However, the significant concentrations of this species as indicated by kernel density analysis using biomass catch data remain largely unprotected (~3% protected; [43]), as do the areas of potential suitable habitat (less than 1% protected) in the core of this species’ predicted distribution (i.e., the Scotian Gulf, Fundian/Northeast Channels and Gulf of Maine (minus areas of model extrapolation), an area equal to ~29,523 km^2^; see Fig 5). Although our model indicated higher probability of occurrence in areas with higher bottom fishing effort, this variable was the least important variable in the model, and examination of its spatial distribution showed few instances of highly localized zones of high effort overlapping with the sponges. The inability of this variable to split the presence and absence data (the determinant of its importance in the model) was reflected by the nearly equal mean fishing effort at the location of presence and absence points (5.08 ± 16.77 and 5.19 ± 16.90 hours, respectively). The low importance of fishing effort in determining the distribution of *V*. *pourtalesi* does not necessarily indicate that sponge grounds outside the conservation areas are not subjected to significant adverse impacts by trawling. Freese and colleagues [96] found that just a single trawl pass through sponge biogenic habitat resulted in an average reduction in sponge densities of 45% compared to untrawled sponge habitat. Future modelling applications examining the cumulative impacts of trawling on the biomass or abundance of *V*. *pourtalesi* might provide further insight into the effects of bottom-tending gear on this species.

Aggregations of sponges typically occur along continental slopes and seamounts [26,28,44,77,97–99] and have been linked to areas of slope that facilitate the propagation of internal tidal waves [97,98] and areas of turbulent mixing at the interface between water masses [30]. Two research groups [97,99] suggested that the predicting factor of their distribution was an increased supply of food and resuspension of organic material caused by the breaking of these waves and turbulent mixing, consistent with the prominence of primary production variables in our predictive model. In contrast, aggregations of the glass sponge *P*. *carpenteri* in the northeast Atlantic occur within a relatively narrow environmental niche [100] and are thought to be associated with areas not directly impacted by enhanced currents, but downstream of them where organic particulate carbon is transported via downslope currents [25].

Similar to *P*. *carpenteri*, the *Vazella* sponge grounds in Emerald Basin occur in areas of low topographic relief, and are strongly linked to areas of warm (> 5°C) and saline (>34) bottom water (Figs 7, 8 and 9). This water mass, identified as Warm Slope Water, originates from the Gulf Stream and represents the dominant flow feature in Emerald Basin. This water mass is characterized by low oxygen (Fig 9) and high nutrient concentrations directly above the sponge grounds compared to the overlying water (Fig 10). Examination of the long-term trends in bottom temperature and salinity (Figs 11 and 12) dating back to 1870 indicated historically high inter-annual variability in the water mass characteristics of Emerald Basin. This variability occurs over multi-decadal timescales and is thought to be associated with a larger than average transport by the Labrador Current off Nova Scotia, which replaces Warm Slope Water with cold, fresh Labrador Slope Water leading to decreased temperatures and salinities within Emerald Basin [51]. One of the first cold periods in the basin on record occurred in the mid-1960’s when bottom temperatures cooled to between 3 and 4.5°C (Fig 11; [51]). Thirty years later, colder temperatures in the basin in 1997 also corresponded to a significant change in offshore slope waters likely caused by a southward flux of the Labrador Current [101]. This multi-decadal variability is consistent with the influence of the Atlantic Multi-Decadal Oscillation, or AMO, a mode of natural variability occurring in the North Atlantic with a period of 60 to 80 years [102]. Overall cooling in sea surface temperature in the North Atlantic from the late 1950’s to the early 1970’s, and overall warming from the 1980’s to the early 2000’s has been attributed to the AMO. The AMO is related to and interacts with the Atlantic Meridional Overturning Circulation (AMOC), which is considered one of the most prominent circulation systems in the Atlantic Ocean and is responsible for heat transport from the South and North Atlantic to the subpolar and polar North Atlantic. Acceleration and deceleration of the AMOC generates a positive (warm) and negative (cold) phase of the AMO, respectively [103]. Recent data suggests that the AMO is currently transitioning to a negative (cold) phase [104]. Such variability indicates that caution should be taken when using species distribution models for long-lived species to define niche requirements under current climatic conditions.

Given that the population of *V*. *pourtalesi* was first documented in Emerald Basin in 1889 [34], we show that this species has persisted in the face of this climatic variability. While the exact temperature and salinity tolerance limits of *V*. *pourtalesi* remain unknown, these results may give insight into how this species will respond to future climate change. In tropical climates, rapid changes in water temperature have shown deleterious effects on the physiology of shallow-water sponges, affecting both their filtration rates and feeding behaviour, as well as their associated microbial component [105], which can comprise between 40 and 60% of the total tissue volume of some species [106]. Similarly, in deep-sea environments, Johnson and colleagues [107] showed that sponge-dominated vulnerable marine ecosystems in North Atlantic areas beyond national jurisdiction (ABNJ) are expected to show deleterious responses to climate change pressures such as increasing temperature. Species distribution models can give useful insight into the potential effects of climate change through the prediction of species’ range expansion or contraction in different climate scenarios, even when the physiological requirements of a species are unknown [108]. Such a process was not undertaken in the current study due to the unavailability of spatially-resolved data for the northwest Atlantic for future climate scenarios. We recommend this as future work in order to understand the impacts of climate change on this species and to develop appropriate measures for its continued conservation.

## Supporting information

S1 Table Presence and absence response data of *V*. *pourtalesi* collected between 1997 and 2017. Data source are DFO's multispecies trawl survey, DFO optical surveys, and commercial catch data from the Fisheries Observer Program (FOP). Note that records from the FOP program do not have a unique trip identifier.
(XLSX)

S1 Fig Correlation matrix based on Spearman’s rank correlation coefficient (*ρ*) between all 63 environmental predictor variables considered for random forest modelling. Circle size indicates the magnitude of the correlation (large circles = highly correlation; small circles indicate lower correlation), and colour indicates direction (blue = positive; red = negative). ann: annual; b: bottom; chl; chlorophyll *a*; cur: current; max: maximum; min: minimum; pp: primary production; ran: range; s: surface; sal: salinity; shr: shear; spr: spring; sum: summer; tmp: temperature; win: winter.
(TIF)

S2 Fig Predicted presence probability of *V*. *pourtalesi* from a random forest model built on *V*. *pourtalesi* presence-absence data, and areas of model extrapolation (i.e., those areas where the environmental data are outside the bounds of those used to train the model) indicated by the grey polygon. Also shown are the presence-absence data used to train the model. Boundary is based on DFO’s Maritimes Region administrative boundary clipped to the 2000-m depth contour. Inset shows the DFO *Vazella* fishery closures and 200 m contour.
(TIF)
