# Supplementary figures and images for: Predicted distribution of the glass sponge Vazella pourtalesi on the Scotian Shelf and its persistence in the face of climatic variability
Source: PLoS One. 2018 Oct 24;13(10):e0205505. doi: 10.1371/journal.pone.0205505 (PMC6200246; doi:10.1371/journal.pone.0205505)

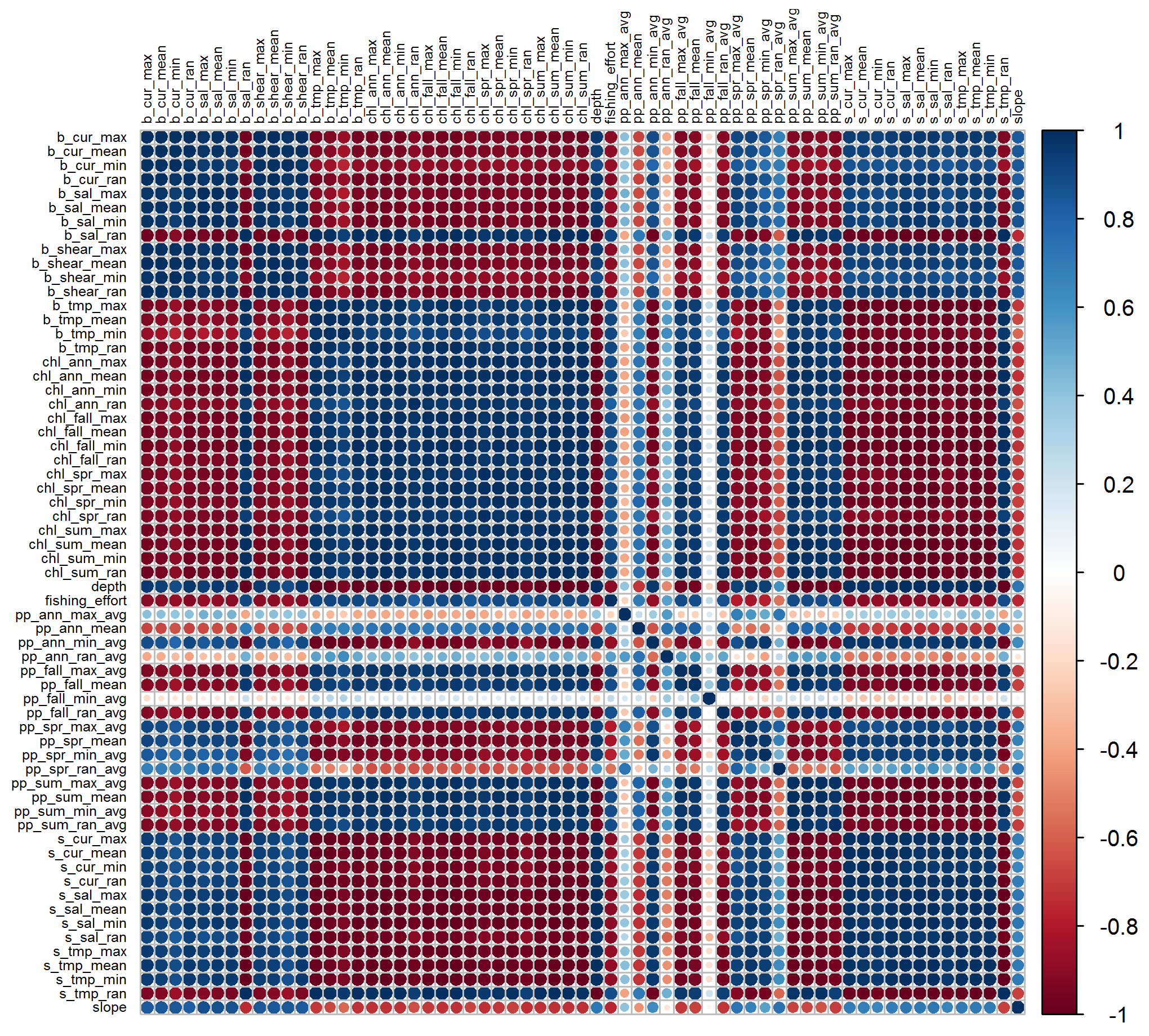

Supplement: S1 Fig — Circle size indicates the magnitude of the correlation (large circles = highly correlation; small circles indicate lower correlation), and colour indicates direction (blue = positive; red = negative). ann: annual; b: bottom; chl; chlorophyll a; cur: current; max: maximum; min: minimum; pp: primary production; ran: range; s: surface; sal: salinity; shr: shear; spr: spring; sum: summer; tmp: temperature; win: winter. (TIF) [file pone.0205505.s002.tif]

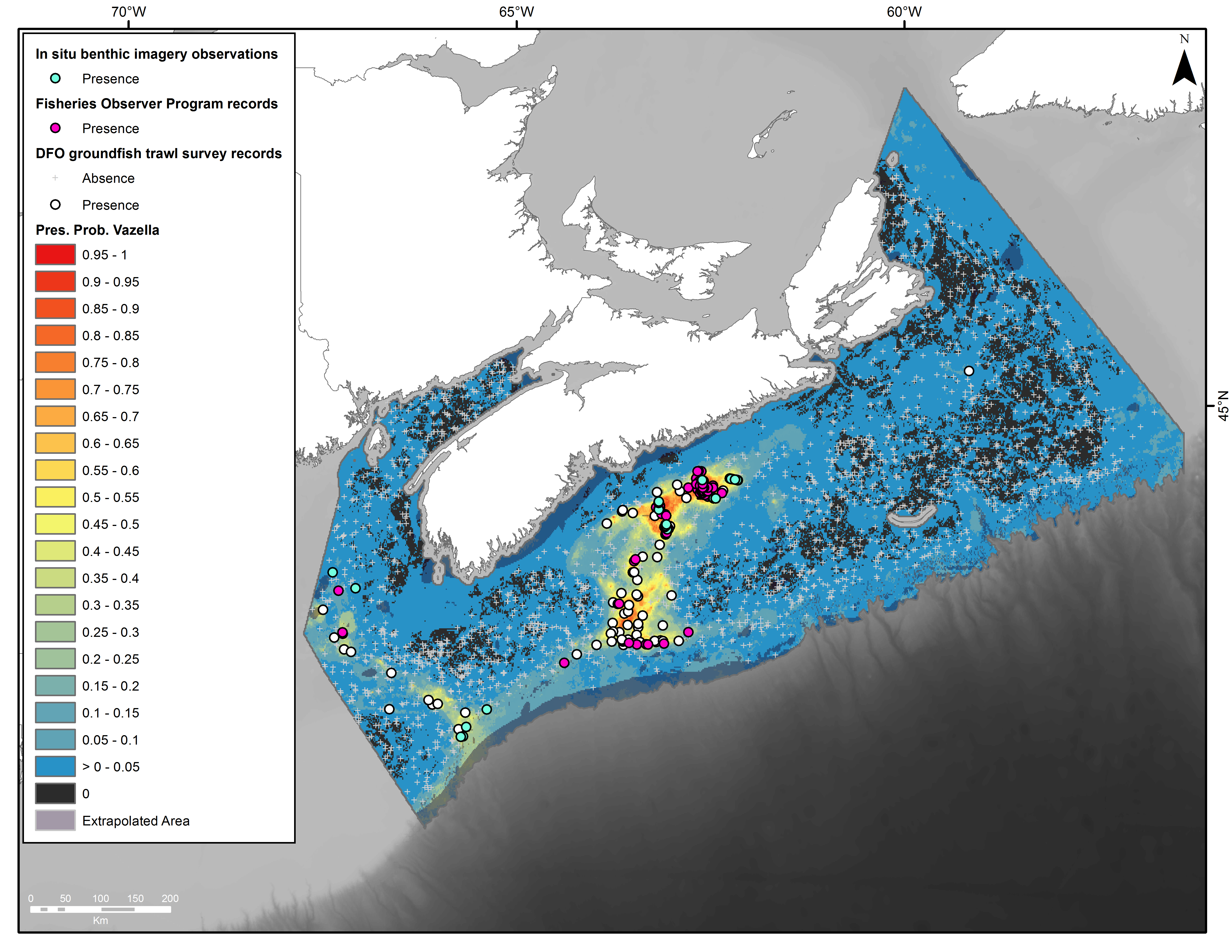

Supplement: S2 Fig — Also shown are the presence-absence data used to train the model. Boundary is based on DFO’s Maritimes Region administrative boundary clipped to the 2000-m depth contour. Inset shows the DFO Vazella fishery closures and 200 m contour. (TIF) [file pone.0205505.s003.tif]
